# Nanoparticles Design for Theranostic Approach in Cancer Disease

**DOI:** 10.3390/cancers14194654

**Published:** 2022-09-24

**Authors:** Barbara Carrese, Gennaro Sanità, Annalisa Lamberti

**Affiliations:** 1 Department of Molecular Medicine and Medical Biotechnology, University of Naples Federico II, 80131 Naples, Italy; 2 Institute of Applied Sciences and Intelligent Systems Unit of Naples, National Research Council, 80131 Naples, Italy

**Keywords:** nanoparticles, theranostics, diagnosis, therapy, contrast agents’ delivery, drug delivery

## Abstract

**Simple Summary:**

Cancer is one of the biggest health problems in the world and its incidence has increased in the last years. Data suggest that cancer-related deaths will hugely raise in the next future. This prevision is directly related to genetic and environmental factors, especially the constant rise of risk factors in developed countries. The demand for new diagnostic and therapeutic strategies is therefore increasing and, in this scenario, theranostics is placed. The possibility to obtain a diagnosis and administer therapy in one platform is a game-changer for medicine. Theranostics, not only allows to overcome some adverse biological effects that may occur when these strategies are employed separately, but also provides the opportunity to save time and money.

**Abstract:**

Presently, there are no conclusive treatments for many types of cancer, mainly due to the advanced phase of the disease at the time of diagnosis and to the side effects of existing therapies. Present diagnostic and therapeutic procedures need to be improved to supply early detection abilities and perform a more specific therapy with reduced systemic toxicity. In this review, improvements in nanotechnology allowing the design of multifunctional nanoparticles for cancer detection, therapy, and monitoring are reported. Nanoparticles, thanks to the nanomaterials they are made of, can be used as contrast agents for various diagnostic techniques such as MRI, optical imaging, and photoacoustic imaging. Furthermore, when used as drug carriers, they can accumulate in tumor tissues through the passive or/and active targeting, protect encapsulated drugs from degradation, raise tumor exposure to chemotherapeutic agents improving treatment effects. In addition, nanocarriers can simultaneously deliver more than one therapeutic agent enhancing the effectiveness of therapy and can co-deliver imaging and therapy agents to provide integration of diagnostics, therapy, and follow-up. Furthermore, the use of nanocarriers allows to use different therapeutic approaches, such as chemotherapy and hyperthermia to exploit synergistic effects. Theranostic approach to diagnose and treat cancer show a great potential to improve human health, however, despite technological advances in this field, the transfer into clinical practice is still a long way off.

## 1. Introduction

Cancer is one of the biggest health problems of our century, its incidence is estimated to increase to about 50% in the next 2 decades because of two main causes: environmental factors (90–95%) and genetics (5–10%) [1,2,3]. The environmental factors contribute to cancer mortality in different ways: 25–30% for smoking, 30–35% for nutrition and obesity, 15–20% for infections, ionizing radiation, stress, and environmental pollutants [3]. Furthermore, the cancer rate is increasing in developing countries because of population aging and cancer-associated lifestyle choices, such as smoking, physical inactivity, and western diet adoption [4]. There are currently no conclusive treatments for a lot of types of cancer, principally for the advanced state of the tumor at the time of diagnosis and for the side effects of the existing therapies and high possibility of relapse [5]. So, early and precise cancer detection represents the key to successfully treat tumors [6]. For this reason, it is of great importance to develop new diagnostic and therapeutic procedures. In particular, the recognition of small tumor masses before the appearance of evident symptoms is of fundamental importance to guarantee the efficacy of the therapy, since most chemotherapeutic drugs are more effective in the early stages of the disease [7,8]. Furthermore, the recognition and elimination of cancer cells at an early stage remains a key goal to avoid the appearance of metastatic sites [9].

In the last years medical research in cancer diagnosis and therapy has grown exponentially and, in this scenario, theranostics is placed. Theranostics is a term derived from a combination of two words: therapeutics and diagnostics. A compound usable for theranostics, approved by Food and Drug Administration (FDA) in 2019 for imaging of somatostatin receptor (SSTR) positive gastroenteropancreatic neuroendocrine tumors, is [^68^Ga]Ga-DOTA-TOC. This radiopharmaceutical compound merges the radionuclide ^68^Ga with DOTA-TOC for specific positron emission tomography (PET) imaging of tumor cells expressing SSTRs [10]. Furthermore, the replacement of ^68^Ga with other radionuclides (^177^Lu or ^90^Y) allows to obtain a therapeutic effect [11].

In recent years, cancer diagnosis and therapy have been improved owing to physicochemical properties of nanoparticles (NPs) and to the possibility to modify NPs surface for a selective targeting [12]. The term “nanoparticles” refers to small particles between 10 and 500 nm made through chemical-physical approaches and consisting of heterogeneous materials (organic, inorganic, biological) [13]. Up to now, the FDA has approved only a few types of NPs for clinical trials, among these the most relevant are liposomes, albumin, iron-based, PLGA-based and silica nanoparticles [14,15]. Furthermore, the exploitation of NPs in medical applications must be subjected to the analysis of specific parameters such as chemical composition, average particles size, shape and morphology, particle size distribution and chemical and physical stability. FDA also approved the administration of nanoparticles in clinics with different modalities: oral delivery for imaging applications; local delivery to transport peptides and other small molecules; topical application to overcome skin barriers; systemic delivery to treat a variety of cancers and other diseases [16].

Nanoparticles show a series of advantages in their use in the biomedical field: have a high modifiable surface to bind molecules of interest through covalent and/or non-covalent interactions, allow the loading of insoluble drugs or contrasts agents (CAs) in considerable quantities increasing solubility and half-life [17]; have chemical-physical properties capable of modulating the release of the drug in response to specific exogenous and/or endogenous conditions (i.e., temperature, pH) [13]. In addition, the use of NPs allows to accurately direct the load to a specific type of target cell to perform precise diagnosis and therapy and represents a good strategy for overcoming the multi drug resistance (MDR), which is the main problem to successful cancer treatment and is decisive to avoid cancer metastasis and relapse [18,19,20].

**Figure 1:**
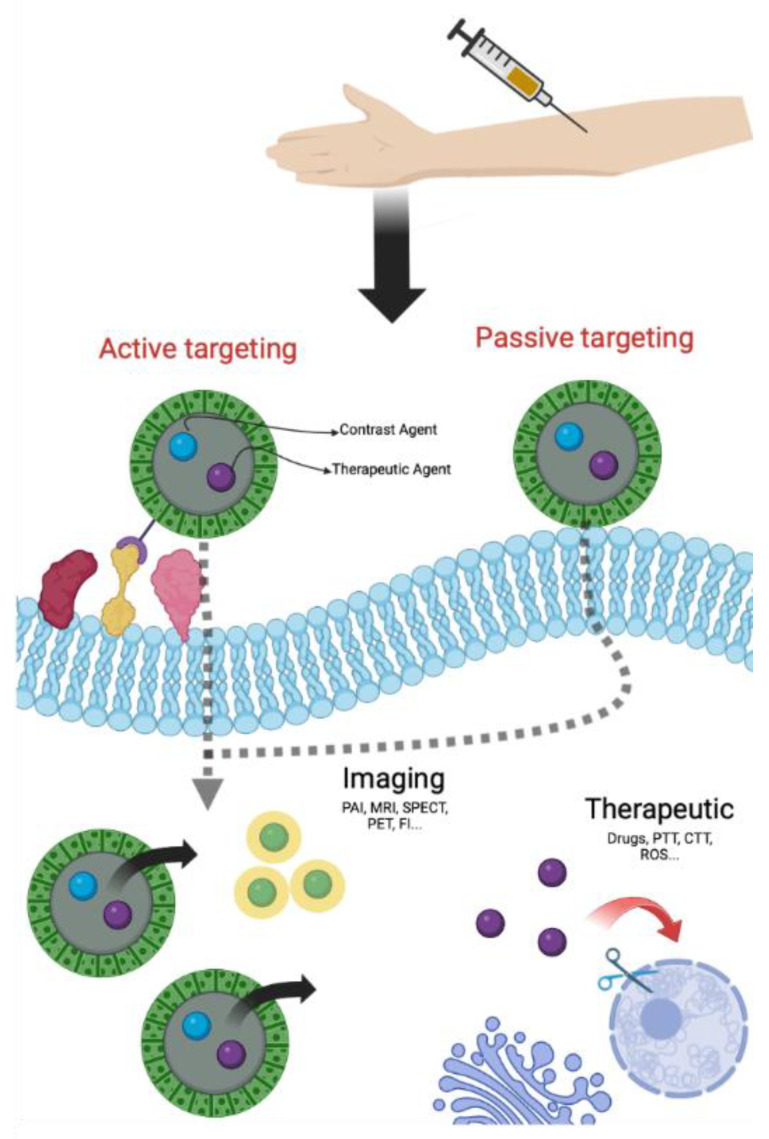
General scheme of theranostic approach (Created with BioRender.com, accessed on 10 September 2022).

NPs can reach tumor cells through two specific pathways, one of passive type and the other of active type. In the passive targeting, NPs accumulate in the tumor site thanks to an irregular neovascularization associated with structural and functional abnormalities and to an altered permeability of tumor blood vessels [21,22]. This phenomenon, known as EPR effect (Enhanced Permeability Retention effect), permits the passive accumulation of NPs in solid tumors and/or metastatic sites owing to their physical properties (size, shape and superficial charge) [23]. The active targeting is a consequence of NPs surface modification with molecules able to bind, with high affinity and specificity, receptors overexpressed on the surface of tumor cells or in the stroma [21,22]. Several ligands, that recognize receptors for nucleoside, glycoproteins, peptides, proteins, transferrin and folate residues, were already reported for active targeting [21]. By using one of these ligands, nanoparticles can penetrate cancer cells via receptor-mediated endocytosis and delivery drugs or CAs (Figure 1) [24]. Furthermore, passive and active targeting can also simultaneously occur.

The synthesis and engineering of innovative nanomaterials exploited to develop NPs for biological applications have contributed in a significant way to increase diagnosis and therapy in cancer disease [25,26], allowing the development of systems to be used in theranostics. The focus of this review is to summarize the different types of NPs exploitable for theranostic applications.

## 2. NPs for Theranostic Applications

The nanomedicine applied to theranostics simultaneously improves detection, spatial targeting as well as tracking of the response to the therapy [27] to realize a more specific personalized medicine [28]. Furthermore, this strategy allows to overcome the typical issues of the conventional diagnostic and therapeutical approaches when used separately and to save time and money.

NPs used for theranostics can be classified based on the size, shape, material and physicochemical properties. In particular, the most important feature of the NPs is their composition because it determines the physicochemical properties of the system, the possibility to functionalize the surface with specific molecules, the type of drug/contrast agents that can be loaded, the therapeutic and diagnostic approach, the stability in biological fluids and the clearance.

The design of NPs for theranostic approach is often based on the combination of two or more inorganic components, two or more organic components or, at least, one of both types of components with specific physio-chemical properties such as optical and electric properties, thermal stability, magnetism and UV-shielding properties. The resulting nanoparticle is not a simple mixture of its components but a nanoplatform with unique features.

**Figure 2:**
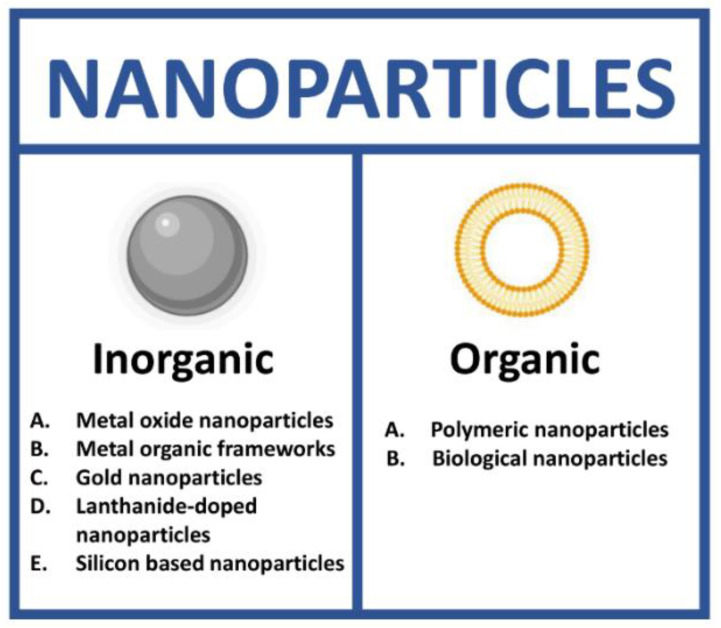
Types of nanoparticles for theranostics. (Created with BioRender.com, accessed on 17 July 2022).

NPs as theranostic tools can be grouped in two different classes: inorganic and organic. In each class, it is possible to identify a high number of subclasses (Figure 2).

### 2.1. Inorganic Nanoparticles

**Figure 3:**
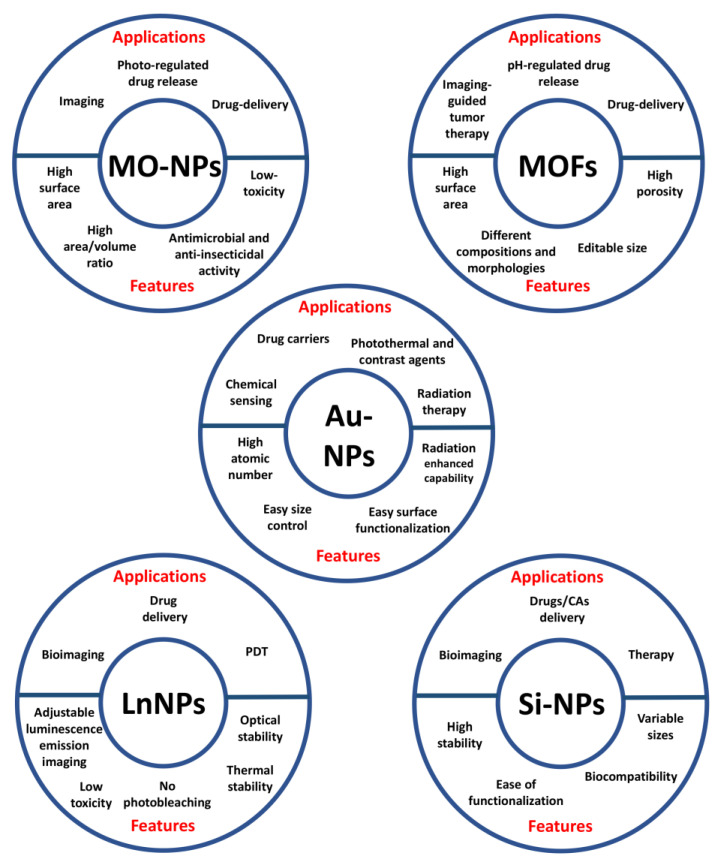
Applications and features of the different types of inorganic nanoparticles.

Inorganic NPs have been extensively described as nanoprobes for theranostic applications thanks to their unique optical, magnetic and electronic properties. This class includes metal oxide (MO), metal organic frameworks (MOFs), gold (AuNPs), lanthanide-doped (LnNPs) and silicon nanoparticles (SiNPs) (Figure 3) [29].

#### 2.1.1. Metal Oxide NPs

Metal and its oxide-based nanoparticles show various physical and chemical properties, such as high surface area and area/volume ratio, low toxicity, antimicrobial and anti-insecticidal activity [30,31,32]. These nanoparticles are extensively studied for photo-regulated drug release, drug delivery and imaging [31,33]. In cancer therapy, metal oxide NPs have shown the capability to specifically kill cancer cells by the generation of reactive oxygen species (ROS) (photocatalytic therapy) [34] and by photo-induced temperature increase (photothermal therapy, PTT). This last is one of the most studied approaches, due to its safety and efficacy [35].

In addition to their intrinsic properties, metal oxide nanoparticles can encapsulate, solubilize or bind a variety of hydrophobic and hydrophilic therapeutic agents (e.g., doxorubicin, DOX), solving stability and solubility problems encountered with the free drugs [30]. Transition metal oxide nanoparticles can be synthesized with a size <100 nm, avoiding rapid elimination by the reticuloendothelial system. Furthermore, MO-NPs surface is often covered with various biocompatible polymers, such as polyethylene glycol (PEG), to reduce cytotoxicity [36].

Among the metal oxide NPs, the most used for theranostic approaches are the iron oxide. These particles are widely exploited both for imaging and for therapeutics by using the metal unique properties. Wang et al. [37] developed superparamagnetic iron oxide NPs (SPIO NPs) modified with an antibody vs Epidermal Growth Factor Receptor (EGFR) to perform active targeting in lung cancer cells (H460). SPIO NPs were tested for magnetic resonance imaging (MRI) and for therapy by magnetic resonance-guided focused ultrasound surgery (MRgFUS). MRgFUS exploits the localization of the tumor masses by MRI to perform US-mediated thermal ablation. One of the limitations of this technique is the US efficacy that exponentially decreases with the depth of the tissue, requiring a high energy that can damage surrounding tissues. To overcome this issue, the authors functionalized SPIO NPs with EGFR to perform active targeting in H460 lung cancer cells and to enhance MRgFUS efficacy. The evaluation of MRI T_2_ imaging performed in vitro showed that bare NPs have a higher T_2_ signal intensity than EGFR-NPs, indicating an increased MRI imaging efficacy due to active uptake receptor-mediated. The imaging (MRI) and therapeutics (MRgFUS ablation) properties of these NPs were also tested in vivo in H460 xenograft mice. The results of MRI and Prussian Blu staining images acquired after different times post-injection (p.i.) showed that functionalized NPs greatly accumulate in the tumor tissues. The Signal-Noise (S/N) ratio analysis showed that after about 4 h p.i the S/N ratio starts to decrease in targeted NPs, compared to the not-targeted ones. This is due to the active accumulation of EGFR modified NPs into tumor mass. The US surgery was tested by using bare NPs compared to EGFR-NPs with different sonication energies (about 50 W and 30 W, respectively). The results showed that, despite the lower power used with EFGR-modified NPs, the reached temperature was about the same as bare NPs (about 80 °C). This result confirms the enhanced therapeutic effect of active targeted NPs compared to not targeted, allowing the use of low energy (a reduction of about 40% in US power) to obtain the same result. The increase of both imaging and therapeutic effect, due to active targeting that enhances NPs uptake in cancer cells, without variation of the chemical-physical properties of the nanoparticles was successfully demonstrated.

Iron oxide NPs were also used in combination with biological molecules, as reported by Quan et al. [38] that developed human serum albumin (HSA) coated iron oxide nanoparticles (HINPs) loaded with doxorubicin (D-HINPs). D-HINPs were designed to perform drug delivery and MRI in 4T1 murine breast cancer xenograft model. These NPs showed a higher cytotoxic effect compared to free DOX and to Doxil^®^, the first FDA-approved nanodrug [39]. The increased cytotoxic effect is due to the high DOX uptake and nucleus localization, as well as the great amount of DOX loaded to D-HINPs (about 7% *w*/*w*). The in vivo DOX uptake in different organs confirmed that D-HINPs accumulates lower in healthy tissues compared to free DOX and Doxil^®^, while their accumulation increased in cancer cells. Furthermore, the analysis of tumor volume showed that D-HINPs have a similar effect of Doxil^®^ preparation. The in vivo MR imaging revealed a significant hypointensity in the tumor site already after 4 h p.i. with a S/N ratio in muscle/tumor of 26% and 42% after 1 h and 4 h p.i., respectively.

In addition to iron oxide, also other metal oxides are investigated for theranostics, such as iridium oxide used by Zhang et al. [40]. The authors describe iridium oxide nanoparticles for in vivo fluorescence imaging, based on the presence of miR-21 in cancer cells, and synergistic chemo and photothermal therapy. The imaging was performed by using a specific DNA guide conjugated with (Cy5)-DNAzyme as contrast agent that produces fluorescence when the guide strand interacts with miR-21. This system is based on the DNAzyme cleavage reaction that releases the fluorescent CA. This mechanism has some advantages: Cy5 is released and activated only in cells with miR-21, the accumulation of Cy5 inside cells increases the fluorescent signal and the cleavage reaction allows the release of the target miR-21, which continues to activate another split DNAzymes precursor. This composite nanosystem also exploits the properties of metals to induce a PT effect and to deliver DOX to realize both photothermal and chemothermal therapy (IO@DNADOX). The bare IO NPs biological assessment was performed in a hepatocellular carcinoma cell line (HepG2) and showed a viability reduction of about 15% after 24 h of incubation with a concentration of 200 µM, suggesting an excellent level of cytocompatibility. The PT and chemothermal effects of IO@DNADOX were evaluated in comparison to IO NPs at 2 different irradiation wavelengths (625 and 808 nm, 1 W/cm^2^ for 1 h). The results showed that both used wavelengths are nontoxic for cells (control) and that 808 nm is more effective compared to 625 nm with both NPs. However, the toxicity of IO@DNADOX was higher (about 80%) than IO NPs (about 60%) under the same conditions. The fluorescence imaging (FI) properties of IO@DNADOX were assessed in HepG2 (high expression of miR-21) and HEK-293 (low expression of miR-21) cell lines, showing a higher Cy5 fluorescence in HepG2 cells compared to HEK-293. The in vivo evaluation was performed in HepG2 tumor-bearing mice with a localization of the fluorescence into tumor after 8 h post-injection. Furthermore, the therapeutic effect was monitored after laser irradiation at 808 nm for 10 min (1 W/cm^2^). The tumor volume was evaluated after different days starting from 15 days p.i. and a tumor growth inhibition of 93.33% compared to control was observed. These results suggest that this nanoprobe could be a good candidate for fluorescence imaging and synergistic chemo- and photo-thermal treatments of tumors, thanks to low cytotoxicity of bare NPs and high tumor growth inhibition of modified NPs.

#### 2.1.2. Metal Organic Frameworks

Metal organic frameworks, also known as porous coordination polymers (PCPs) or coordination networks, include a class of organic-inorganic hybrid porous materials formed by organic ligands, cluster nodes and metal ions, linked through coordination bonds that allow the degradation of PCPs [41]. Furthermore, MOFs show unique properties, such as editable size, high porosity, different compositions and morphologies, high surface areas and large porosities to load small organic molecules and macromolecules [42]. Nowadays, nanoscale MOFs (NMOFs) are explored for a variety of application, including drug delivery and imaging-guided tumor therapy, such as MRI, photoacoustic imaging (PAI), PET and FI, representing excellent materials for theranostic purpose [43].

Zhao and its collaborators [44] designed MOF-based Fe_3_O_4_@UiO-66 core–shell composites loaded with DOX to simultaneously perform drug delivery and MRI. The UiO-66 shell was used to encapsule the drug, while the Fe_3_O_4_ core acts as a MR contrast agent with a high transverse relaxivity of about 255.87 mM^−1^ s^−1^. The MOFs showed an ultrahigh DOX loading capacity of 2.0 mg DOX per mg composites (66.3% *w*/*w*), which is almost the highest DOX payload compared to other MOFs. They tested Fe_3_O_4_@UiO-66 composites cytotoxicity in a cervical cancer cell line (HeLa) and no obvious toxicity was observed (approximately 100% cell viability), even at a concentration of 500 μg/mL. Contrary, DOX loaded MOF exhibited a dose dependent anticancer activity against HeLa cells. Furthermore, they performed T_2_-weighted MR images of the cells incubated with different concentrations of Fe_3_O_4_@UiO-66 composites (from 0 to 200 μg/mL). Tumors cells became much darker with increasing concentrations of Fe_3_O_4_@UiO-66, demonstrating the MOF capability as a T_2_-weighted MR contrast agent for in vitro MR imaging. The use of Fe_3_O_4_@UiO-66 composites for in vivo MR imaging in HeLa-tumor bearing mice was also evaluated, showing a darkening effect in the tumor area after 1 h post-injection. The in vivo anti-tumor efficacy was tested treating mice with Fe_3_O_4_@UiO-66 composites and a growth tumor inhibition was observed after 7 and 30 days from administration.

An approach to perform active targeting by using MOFs is reported by Mukherjee et al. [45]. Smart magnetic nanocomposite NaGdF_4_:Yb/Er@MIL-53(Fe) covered with folic acid (FA), that allows imaging-guided targeted drug delivery, were designed. These nanocomposites showed a drug loading efficacy of 16% and a pH dependent drug release of about 80% after 48 h at pH~5.2, due to the dissolution of this nanocomplex in the acidic medium. NaGdF_4_:Yb/Er@MIL-53(Fe)/FA nanocomposites are noncytotoxic in both cancer (B16−F10) and normal (HEK293) cells, indeed above 80% of cell viability was observed up to 5 µg/mL. Cytotoxicity effect of DOX-loaded NaGdF_4_:Yb/Er@MIL-53(Fe)/FA was then evaluated in both cell lines, showing a dose-dependent cytotoxicity only in cancer cells, but not in normal cells, probably due to the presence of folate-receptor-mediated endocytosis of the drug-loaded MOFs. Moreover, the nanocomposite showed good paramagnetic properties with a saturation magnetization value of 1.4 emu/g. Owing to this feature, they could be useful as platform for both T_1_ and T_2_ MRI contrast agents in the future.

#### 2.1.3. Gold NPs

Gold NPs are promising carriers for drugs and are photothermal active particles for cancer imaging and therapy. Indeed, AuNPs show anticancer effect owing to the physicochemical interactions of gold atoms with chemical functional groups of intracellular macromolecules such as proteins and nucleic acids [46,47]. Furthermore, gold nanoparticles are ideal nanocarriers for radiation therapy, thanks to their high atomic number and radiation-enhancing capability, ease of size control and surface functionalization [48]. The main disadvantage of gold NPs is their high cost, which restricts a widespread use [47].

Mangadlao et al. [49] reported gold nanoparticles loaded with fluorescent photodynamic therapy drug (Pc4) modified with prostate-specific membrane antigen (PSMA-1) (AuNP-5kPEG-PSMA-1-Pc4). The biological in vitro assessment demonstrated a higher uptake in PSMA-positive prostate cells (PC3pip) compared to PSMA-negative prostate cells (PC3flu), confirming the active targeting, and allowing more cell ablation in PC3pip (4.9% of viability) compared to PC3flu cells (37.1% of viability), under light exposure (>500 nm) at different doses (from 0 to 1 J). In xenograft mice models, these nanoparticles (0.07 mg/kg), after 3 h p.i., accumulate in PC3pip tumor showing a fluorescence signal about 10-fold higher than PC3flu tumor. For therapeutic evaluation the tumor was irradiated with a wavelength of 672 nm (150 and 300 J/cm^2^) and, after 14 days, a significant tumor growth inhibition was observed, demonstrating that these nanoparticles could represent surgical guidance for tumor resection and therapeutic approach when surgery is insufficient.

Gold NPs exploited for theranostic applications are also described by Yoon et al. [50]. The authors realized smart gold nanoparticle-stabilized microbubbles (SAuMBs) constituted of a gas-filled core and a shell containing smart gold nanoparticles (SAuNPs). The gas core allows the detection of tumors by ultrasound and enables the administration of SAuNPs by sonoporation. In tumors, SAuNPs aggregate allowing both PAI and PTT. The biological effects of SAuMBs (0.1–5 nM) were evaluated in vitro in a glioblastoma cell line (U-87 MG). No cytotoxicity was observed in cells treated with SAuMBs alone and in combination with US. On the contrary, the cell viability decreased to approximately 25% in cultures treated with SAuMBs, US, and laser irradiation at 671 nm for 5 min, owing to photothermal effect. The properties of these nanoparticles were evaluated also in vivo by using U-87MG xenograft mice. After injection of SAuMBs (10 nM), SAuNPs were observed only in tumors of mice subjected to US treatment, and fluorescence due to SAuNPs was observable for at least 24 h. The PTT effect of SAuMBs on cancer tissues was evaluated, after injection and US treatment, irradiating with a laser at 671 nm with an intensity of 0.5 W/cm^−2^ for 8 min, and a significant decrease in tumor mass was observed already 4 days after treatment. The authors show a good strategy for the treatment of cancer by using SAuNPs to monitor and treat cancer through PAI and PTT, respectively, and SAuMBs as theranostic agent.

#### 2.1.4. Lanthanide-Doped NPs

Lanthanides are a group of 15 metal elements with atomic numbers between 57 and 71 and with chemical properties very similar to lanthanum, the element from which they acquire their name.

Lanthanide-doped nanoparticles can be used for different applications such as bioimaging, drug delivery and photodynamic therapy (PDT) [51,52]. The principal feature of LnNPs is the adjustable luminescence emission and the good optical and thermal stability, since the photoluminescent signal has a long lifetime (from microseconds to milliseconds), low toxicity, and no photobleaching [51,53]. LnNPs are also known as upconverting luminescent nanoparticles (UCLNPs) thanks to their extensive properties of up-conversion luminescence (UCL) under Near InfraRed-I (NIR-I) light [54] and Near InfraRed-II (NIR-II) light [55].

Zhou et al. [56] developed Lipo-FNPs by loading carboxyl-containing ferrocene derivative on the UCLNPs (NaYF_4_:Yb, Tm@NaYF_4_:Eu) surface. Successively, UCLNPs were encapsulated into liposomes. This system was tested to perform UCL imaging and efficient NIR promoted photo-Fenton therapy of hypoxic tumors. As cell models, a gastric adenocarcinoma cell line (AGS), a liver cancer cell line (HepG2), a cervical cancer cell line (HeLa), and HUVEC healthy cells were used. This nano-platform merges the high biocompatibility and loading capacity of liposomes with the unique properties of lanthanide-doped materials. NIR laser irradiation at 980 nm promotes Fenton-like reaction producing toxic OH^•^ and FNPs^+^ with the decrease of H_2_O_2_ concentration. Next, FNPs^+^ are reduced to FNPs by GSH. The H_2_O_2_/GSH depletion brings intracellular redox homeostasis alteration allowing the anticancer therapy. A significant light-induced toxicity was observed at about 30 µg/mL only in cancer cell lines (IC_50_ value: HeLa 34,64 µg/mL, AGS 22.54 µg/mL and HepG2 28.01 µg/mL). The UCL signal for imaging was evaluated both in vitro and in vivo in AGS cells and in AGS tumor-bearing mice. After laser irradiation at 980 nm, the fluorescence signal was well detected both in cells and in tumor mass in mice. The in vitro analysis showed a good red fluorescence signal only inside cells, confirming NPs uptake with a good contrast. The in vivo experiments revealed a high Lipo-NPs uptake only in tumor mass after 6 h p.i., with a stable signal up to 24 h and a tumor growth suppression of about 80%, with no weight loss in treated mice and very low damage to healthy tissues. Interestingly, the therapeutic effect on tumor region of this nanoplatform is due to overexpression of H_2_O_2_ in acidic tumor microenvironment, enabling excellent specificity and minimum damage to healthy tissues.

Since one of the most important features of “light-induced effects” in nano theranostic is the capability of light to penetrate tissues with low scattering and adsorption phenomena, various wavelengths were tested to ensure a good depth of the signal. Li et al. [55] realized a high-performance theranostic nanoplatform composed of core-shell structured nanorods (NaLuF_4_) conjugated with polydopamine (NRs@PDA). The authors explored NIR-II tumor-associated blood vessels imaging at a wavelength of 1500 nm and PTT, both in vitro in a cervical cancer cell line (HeLa) and in vivo in Lewis lung cancer (LLC) tumor-bearing and in colon HCT-116 xenograft model. NRs@PDA NPs cytotoxicity was assessed in vitro, and up to 1 mg/mL low levels of toxicity (about 13%) was observed. NIR-II imaging in LLC tumor-bearing and normal mice showed a good NIR-II fluorescent signal distribution in tumor vessels, allowing to measure their thickness. This imaging technique highlighted that the vessels associated with the tumor were wider (0.68 mm) than the normal ones (0.49 mm), allowing the identification of the neovascular formations and the detection of the anomalies of blood vessels. Furthermore, this nanoplatform was exploited to perform brain/abdomen vessel imaging with a high spatial resolution (45.15 μm). NRs@PDA NPs were also tested as PTT agent by laser irradiation at 808 nm, showing significant tumor growth inhibition only when NRs@PDA NPs and 808 nm light were simultaneously used. Furthermore, in vitro and in vivo experiments confirm that the designed nanoplatform has high photothermal conversion efficiency (40.18%) for PTT ablation with a high quantum yield of photoconversion (1.37%). This nanosystem, with surprising physical properties, is an excellent example of how the combination of different materials can give very promising results.

#### 2.1.5. Silicon Based NPs

Silicon-based NPs are particles composed of crystalline or amorph silica and can be synthesized with variable sizes (from 2 nm up to 500 nm) by using different chemical approaches. SiNPs are widely used to realize nanoplatforms for drugs/CAs delivery owing to their high stability, ease of functionalization and biocompatibility.

Silvestri et al. developed a new kind of hybrid nanoprobe, formed by an external shell of silicon and a melanin-like compound (5,6-dihydroxyindole-2-carboxylic acid, DHICA), and an internal cluster of metallic silver (MelaSil_Ag NPs). These hybrid NPs, thanks to the presence of DHICA as CA, showed promising PA properties by using harmless radiation. To selectively target tumor cells via HSA–SPARC interaction, MelaSil_Ag NPs were functionalized with HSA. To evaluate the effect of HSA functionalization on NPs cytotoxicity, bare and HSA modified NPs were tested in vitro in a breast cancer cell line (HS578T) and in a normal immortalized breast cell line (MCF10a) at increasing concentrations (from 0 to 100 μg/mL). The results clearly showed nontoxicity of HSA-NPs compared to bare NPs. The PAI study demonstrated the increase of the photostability of MelaSil_Ag-HSA NPs compared to bare NPs. Furthermore, the intensity of PA signal after NPs uptake inside cells showed values up to four-time stronger compared to the NPs free cells. These NPs were successively loaded with DOX (MelaSil_Ag-HSA@DOX NPs) to perform chemo- and photo-thermal therapy. HS578T cells were incubated with increasing concentrations of free DOX (0.65 μM, 1.3 μM, and 2.6 μM) and DOX-loaded NPs (30, 60 and 120 μg/mL). The results indicate that, in all tested conditions, MelaSil_Ag-HSA@DOX NPs were more toxic than the free drug (13% vs. 53% after 72 h at the lowest drug concentration). To explore the toxicity of the synergistic effect of NPs administration and laser irradiation, cells were incubated at different times with increasing concentrations of DOX- NPs or HSA-NPs and irradiated with a wavelength of 808 nm (3 W/cm^2^) for 5 min. After irradiation, the cytotoxicity was higher than dark conditions already at 6 h of treatment at the lowest DOX concentration. The combined administration of chemo- and phototermal components is a promising approach to reduce time of treatment and concentration of DOX [20,57,58].

Ferreira et al. [59] developed ultrasmall silicon-based nanoparticles to effectively exploit the EPR effect. The ultrasmall porous silica nanoparticles (UPSN) were covered with DOTA (1,4,7,10-tetraazacyclododecane-1,4,7,10-tetra-acetic acid), a chelator that binds the radionuclides isotopic pair ^90^ Y and ^86^Y to perform radiotherapy and PET imaging, respectively (^90/86^Y-DOTA-UPSN). To assess the efficacy of this nanoplatform, PET imaging in vivo experiments were performed by using 4T1 tumor-bearing mice and images were acquired at different times p.i. of ^86^Y-DOTA-UPSN (2–3 MBq). The results showed a time progressive accumulation in tumor mass with a maximum tumor/muscle ratio at 48 h. Contrary, healthy organs showed a very low nanoparticles accumulation, with a very slight signal. This is a clear indication of the passive targeting efficacy due to the ^86^Y-DOTA-UPSN very small size. The in vivo therapeutic effect of ^90^Y-DOTA-UPSN (5.5 MBq) was assessed in the same model. The tumor growth was significantly inhibited starting from day 1 p.i. compared with controls (PBS, UPSN and ^90^Y-DOTA alone), with a tumor regression of 30% after 11 days. Furthermore, the survival rate of ^90^Y-DOTA-UPSN treated mice significantly increased compared to controls. These results suggest that ultra-small SiNPs could be used to perform passive targeting with high efficacy, and the combination of these NPs with radionuclides could be exploited for theranostics.

### 2.2. Organic Nanoparticles

**Figure 4:**
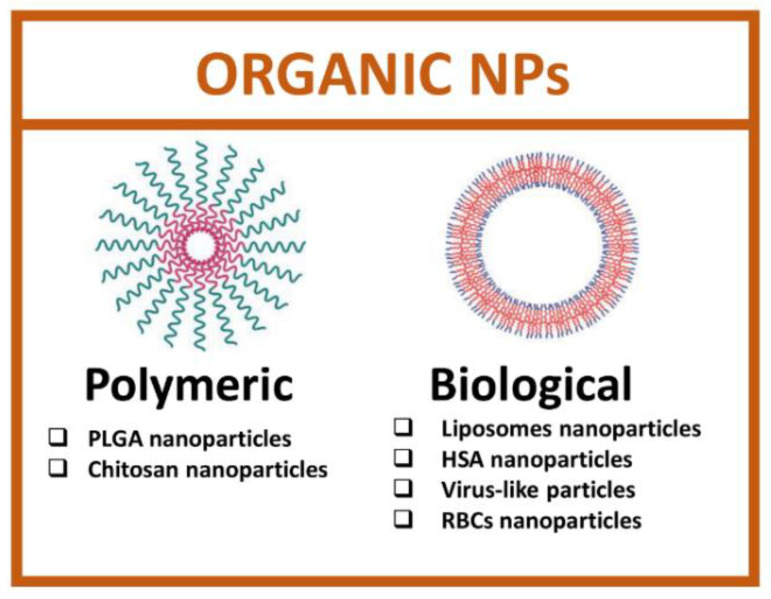
Types of organic nanoparticles. (Created with BioRender.com, accessed on 17 July 2022).

Organic nanoparticles are developed starting from biological macromolecules, such as proteins, lipids, or viral capsid, and synthetic organic molecules [60]. The main advantage of using organic nanoparticles is the total safety, biocompatibility and ease of functionalization due to the presence of many functional groups. Organic nanoparticles comprise a heterogeneous set of categories and, among these, the most studied are polymeric and biological NPs (Figure 4).

#### 2.2.1. Polymeric Nanoparticles

Polymeric nanoparticles are composed of natural or synthetic polymers [61] that can be loaded with active compounds inside NPs core or on the surface by chemical modification or by chemisorption [62]. Moreover, polymeric NPs loaded with drugs can release their cargo by diffusion, polymer matrix swelling, polymer erosion, or degradation. Thanks to their excellent endocytosis efficiency, passive tumor-targeting and high encapsulation efficiency, polymeric NPs are preferred to other nanosystems. Furthermore, polymeric NPs are degraded into nontoxic compounds [63]. Among the polymers used to realize theranostic NPs, the most interesting are poly D, L-lactide-co-glycolide (PLGA) and chitosan.

PLGA polymer is biocompatible and biodegradable and allows to obtain nanoparticles for controlled drug release and to target cancer cells by passive targeting. The ease of surface functionalization with folate, antibodies or peptides can be also exploited to perform active delivery. Furthermore, PLGA NPs can be easily hydrolyzed in their monomers (i.e., lactic acid or glycolic acid) and eliminated through metabolic pathways in the human body [64].

Wang et al. [65] reported a nanoplatform composed by PLGA NPs covered with manganese dioxide (MnO_2_) ultrathin nanofilms, for MRI and X-ray computed tomography (CT), and loaded with gold nanorods (AuNRs), for radio frequency (RF) hyperthermia, and with docetaxel (DTX) for chemotherapy. The final nano construct (PLGA/AuNR/DTX@MnO_2_) was assessed for theranostic applications in a breast cancer cell line (MCF-7) for in vitro experiments, and in a xenograft mice model, obtained by intravenous injection of murine fibrosarcoma cancer cells (S180), for in vivo toxicity evaluation, and for dual modal imaging (MRI and X-ray CT). To investigate cytotoxic effects in vitro, cell viability by using NPs with AuNRs from 0.5 to 2 μg/mL and DTX from 10 to 40 μg/mL in different ratios, with or without RF treatment, was evaluated. Without RF treatment, AuNR@MnO2 showed negligible toxicity and there was no significant difference among different ratios. After RF treatment, the cell viability of each ratio decreased for the presence of Au by PT effect. In in vivo studies, anti-tumor effect was evaluated by measuring the relative tumor volume and the body weights for 10 days. The results indicated that the combination of AuNRs-induced RF hyperthermia and DTX-induced chemotherapy could be used to reach efficient tumor inhibition. Furthermore, imaging studies showed that the biodistribution of PLGA/AuNR/DTX@MnO_2_ in the tumor site was maximum after 4 h post-injection. NPs containing Au are always used for NIR-PTT, however NIR laser irradiation has low tissue penetration and causes skin burning. The use of RF could represent a good strategy to overcome these issues.

PLGA-based NPs were also investigated for PAI and PTT as reported by Li et al. [26]. The authors realized a copolymeric Polyethylene Glycol−PLGA nanosystem loaded with near-infrared croconaine (infrared photoacoustic and pH-dependent light adsorbing dye) exploitable for in vivo multiplexed PA imaging and pH-responsive photothermal therapy. Furthermore, the authors covered the NPs with iRGD peptide (a cyclic peptide composed of 9 amino acids specific for cell penetration and molecular mimetics) to perform active targeting [66]. The photothermal conversion efficiency (η) of NPs was calculated in buffer solutions at three different pH (5.8, 6.5 and 7.4). The intensity of the PA signal after 3 h of incubation was 4.0-fold higher at pH 5.8 compared to pH 7.4. In vitro, the determination of the cytotoxicity of PLGA-NPs (0–60 μg/mL), with and without laser irradiation, was performed by using breast cancer cells (MDA-MB-231). Without laser irradiation, the IC_50_ value is 60.2 μg/mL after 24 h of treatment and decreases to 11.2 μg/mL after laser irradiation at 808 nm (1.0 W/cm^2^ for 5 min), demonstrating an efficient photothermal therapeutic effect. PA imaging of NPs (50 μg/mL) was also evaluated in breast cancer cells for different times. The PA signal after laser irradiation at 815 nm was strong within 24 h, indicating that the signal intensity increased with the incubation time. These NPs were tested also in vivo for PAI and PTT, in xenograft mice models obtained through MDA-MB-231 cells injection in nude mice. PA images were acquired for increasing times by using NPs at 5 mg/kg and the PA signal increased quickly, with the maximum peak after 22 h post-injection. A weak signal remained even after 72 h p.i., indicating an extended NPs circulation time. For PTT, mice were irradiated at 808 nm (1.5 W/cm^2^) and, after 24 h from laser irradiation, tumor mice exhibited a significant tumor regression when compared to control group. The results demonstrated that in vivo NPs showed the ability to inhibit tumor growth with no acute toxicity to major organs after a single dose of intravenous injection.

The synthesis of PLGA-based NPs, by using organic and inorganic materials, is also reported by Dong et al. [67]. The authors exploited PLGA NPs loaded with perfluorooctyl bromide (PFOB) and with superparamagnetic iron oxide particles and coated with gold nano shell. Furthermore, the resulting nanostructure was conjugated with an anti-Her2 antibody (Her2-GPH NPs). These NPs were exploited for dual-modal imaging by US/MRI and for PTT. The US properties are possible owing to the presence of PFBO, while MR and PT properties are due to gold and iron oxide. After 10 min of NIR laser irradiation (808 nm, 1 W/cm^2^), the thermal imaging color of different concentrations of Her2-GPH NPs solution changed, revealing a rise of temperature with NPs increasing concentrations (50–200 μg/mL). In addition, the signal intensity of T_2_-weighted MRI slowly decreased with the increase of the iron concentration, confirming that Her2-GPH NPs can be used for T_2_-weighted MR. The Her-2 dependent active targeting was confirmed in vitro in Her-2 positive breast cancer cells (SKBR3) compared to Her-2 negative cell line (MDA-MB-231). US and MR imaging was assessed in SKBR3 cells, confirming a high signal/noise ratio and contrast in both the imaging techniques. Indeed, in US imaging, the gray-scale value of SKBR3 cells treated with Her2-GPH NPs was significantly higher compared to control. For MRI, SKBR3 cells treated with Her2-GPH NPs exhibited noticeable negative contrast enhancement and showed a signal decrease (48%) compared to non-treated cells. Furthermore, Her2-GPH NPs-treated SKBR3 cells showed a higher signal intensity reduction compared to MDA-MB-231 cells, which could be used to distinguish the two cell lines. The PT effect was evaluated by incubating both SKBR3 cell culture alone and SKBR3 + MDA-MB-231 co-culture (at different proportions of SKBR3 to simulate the heterogeneous expression of Her2 in breast tumor tissues) with NPs at increasing concentration (0–200 μg/mL), in presence or in absence of NIR laser irradiation. No significant cytotoxicity (10%) was observed in SKBR3 cells alone treated in absence of NIR laser irradiation compared to irradiated cells (80% at 200 μg/mL). When SKBR3 were co-cultured with MDA-MB-231 cells, the total cell viability after irradiation decreased with the increasing proportions of SKBR3 cells. Despite the promising results obtained by authors in vitro for dual-modal imaging by US/MRI, in vivo studies are needed.

Chitosan is a natural pH-sensitive polymer with several properties, such as biocompatibility and biodegradability and high cellular uptake. Furthermore, the advantage of using chitosan is that this polymer can easily be converted into nanoparticles forming a stable polyplex with both CAs and therapeutic agents in a single step. Gholami et al. [68] realized a chitosan-based nanoplatform, by using poly-L-arginine-chitosan-triphosphate matrix (ACSD) as carrier for doxorubicin (~12% loading efficiency) and superparamagnetic iron oxide nanoparticles (SPIONs). These nanoparticles showed a pH-dependent DOX release for specific therapy approach in cancer environment and MRI properties. NPs biological assessment, imaging and therapeutic efficacy were tested in vitro in glioma cell line (C6). Cells were treated with NPs at increasing concentrations (1.9 to 500 μg/mL) and the corresponding free DOX, showing that, when DOX is delivered by NPs, its IC_50_ decreases of about 40-fold respect to free drug. MRI properties were evaluated in agar phantom displaying a high efficacy and an increase in signal intensity when the iron concentration increases (0–0.15 mM). MRI studies in C6 cells were performed by using NPs at 250 μg/mL and T_2_-MRI images showed the presence of darker signal correlated to the position of labelled cells in comparison with non labelled cells.

Chitosan-based NPs were also investigated to deliver therapeutic nucleic acid. Sahoo et al. [69] realized a chitosan-based NPs loaded with highly fluorescent trifunctional Au nanoclusters (emitting in red, blue and green wavelengths), that provide useful optical imaging, and a suicide gene (coding for cytosine deaminase uracil phosphoribosyltransferase, CD−UPRT) that triggers apoptosis in cervical cancer cells (HeLa). To test these NPs as intracellular probes, HeLa cells were incubated with NPs (50 μg/mL) for 4 h, followed by microscopic imaging. Images revealed emission in all three colors both in nuclei and cytoplasm, indicating that NPs are useful to perform clear images. Furthermore, these NPs are nontoxic for mammalian cells, even at 100 μg/mL (5% of mortality) and could be used for imaging in vivo. These nanonocarriers, thanks to their fluorescent properties, could help to evaluate suicide genes delivery, opening new horizons in nanotechnology-based drug delivery. Both reported studies on chitosan-based nanoparticles lack of analysis in vivo that are required for biodistribution and systemic toxicity evaluation.

#### 2.2.2. Biological Nanoparticles

Biological NPs comprise a series of nanoparticles composed of biological macromolecules (such as lipids and proteins), viral capsid or red blood cell membranes. These NPs show uniform structures, low toxicity, ability to escape the immune system and ease of modification [70].

Lipid-based NPs (liposomes) were the first approved by FDA in 1995 as nanomedicine platform to treat AIDS-related Kaposi’s sarcoma in the formulation of Doxil^®^ (PEGylated liposome loaded with doxorubicin) [57]. Liposomes are spherical vesicles composed of a lipid bilayer consisting of synthetic or natural phospholipids with a diameter of about 100 nm. For theranostic purposes, liposomes can be loaded with a variety of drugs and CAs [71].

Feng et al. [72] developed a liposome-based tool loaded with a hypoxia-activated prodrug (AQ4N) inside the aqueous cavity and a hydrophobic hexadecylamine conjugated chlorin e6 (hCe6), a photosensitizer, in the hydrophobic bilayer. The high loading capacity of liposomes was exploited to load a high amount of both AQ4N prodrug and 64Cu-hCe6 photosensitizer (10.8% and 85% *w*/*w*, respectively). The cellular internalization was performed in murine breast cancer cells (4T1) incubated with AQ4N-hCe6-liposomes, hCe6-liposome, and free AQ4N. Cells incubated with AQ4N-hCe6-liposomes showed strong fluorescence of both AQ4N and Ce6, indicating clearly that liposomes can enhance both Ce6 and AQ4N cellular uptake. The hypoxia-induced toxicity was tested in 4T1 cells comparing normoxic and hypoxic conditions. The results showed a IC_50_ of AQ4N at 37.8 µM in normoxic condition vs 2 µM in hypoxic condition. Furthermore, the photodynamic features of AQ4N-hCe6-liposome were tested in vitro after laser irradiation at 660 nm. The cytotoxicity due to Ce6 was observed already at a concentration of 1 µM, with 100% of toxicity at about 5 µM. Furthermore, the photodynamic therapeutic effect was assessed also in vivo in tumor-bearing Balb/c mice injected with breast cancer cells (4T1), where a significative tumor growth inhibition was observed. To perform PET imaging, AQ4N-hCe6-liposomes were modified with ^64^Cu isotope and injected in mice. After 4 h p.i., a good accumulation in tumor site was observed (4.9 ± 0.75% ID g^−1^) by using PET fluorescence and photoacoustic techniques with a tumor/muscle signal ratio of about 5. This nanoplatform is a promising candidate for future clinical translation due to excellent biocompatibility and versatility as CA that could facilitate tumor imaging and real-time in vivo monitoring.

Another example of liposomes exploitation for theranostics is reported by Prasad et al. [73]. The authors realized graphene oxide flakes fortified Liposome (GOF-Lipo) bioconjugated with folic acid (FA), for active targeting, and loaded with DOX (DOX-GOF-Lipo-FA). The graphene flakes work such as a stabilizer enhancing the drug loading capacity of the NPs, preventing the drug release in the extracellular environment, and working as PT agent. This nanoplatform was assessed for photo and chemothermal therapy and NIR fluorescence imaging in vitro in breast cancer cells (MDA-MB-231 and 4T1) and in vivo in 4T1 breast tumor-bearing female Balb/c mice. The in vitro assessment showed high biocompatibility of GOF-Lipo-FA (about 90% at 2 mg/mL) in normal cells (L929). The NIR-mediated effect on cellular toxicity, by irradiating with a wavelength of 808 nm, was assessed in both MDA-MB-231 and 4T1 cell lines. The combined effect of DOX release and heating induced a toxicity up to about 90% in both cell lines. In vivo results showed, through NIR light treatments, specific localization of DOX-GOF-Lipo-FA in breast tumor after 24 h p.i. due to FA targeting. Furthermore, PTT/CTT GO-mediated effects were observed with a temperature increase up to about 50 °C in the tumor site, compared to 36 °C in untreated mice. The tumor growth was highly inhibited with DOX-GOF-Lipo-FA (tumor mass of about 0.13 g) compared to free drug (about 0.22 g), demonstrating the positive effect of the PTT/CTT combination. Most significant features of these nanoparticles are the specific biodistribution in breast tumor and a good tumor regression by using a single dose.

Among the proteins exploited to realize biological NPs, the most used are bovine serum albumin (BSA) and HSA. These proteins have several advantages such as non-immunogenicity and nontoxicity, ease of functionalization, capability to bind several drugs, low cost, and can be used both to realize HSA-NPs and to functionalize the surface of other types of NPs. Furthermore, HSA binds glycoproteins Gp60, Gp30, and Gp18 and the secreted protein acidic and rich in cysteine (SPARC), allowing a specific NPs active targeted delivery [20]. It has been supposed that the tumor increase uptake of nab-paclitaxel (Abraxane^®^) occurs by this SPARC mediated pathway [74]. For these reasons, HSA-NPs are a promising tool for specific delivery of drugs and CAs to cancer cells [60].

Zhao et al. [75] developed an albumin-based photothermal therapy nanoplatform by modifying HSA with a P-selectin-targeting peptide (PSN peptide) and with IR780 (photosensitizer and fluorescent CA). Furthermore, HSA NPs were loaded with paclitaxel (PTX), (PSN-HSA-PTX-IR780 NPs). This work exploits an “enhancing accumulation effect” of nanoparticles due to hyperthermal injury. In fact, the thermal damage increases tumor-infiltrating platelets, which recruited more nanoparticles into the tumor, resulting in an enhancement of imaging signal and therapy efficacy. In vitro (4T1 breast cancer cells) and in vivo (tumor-bearing Balb/c mice injected with 4T1 cells), experiments to assess the cytotoxicity, uptake, intracellular traffic and biodistribution, PTX accumulation, targeting and antimetastatic properties were performed. The in vitro results, assessed with SEM, fluorescence microscopy and flow cytometry showed a preferential accumulation of PSN modified NPs compared to the bare one (uptake enhancement of 60%). Furthermore, in vitro experiments to assess cytotoxicity induced by laser irradiation at 808 nm for 5′ were performed by using free PTX-IR780, HSA- PTX-IR780 and PSN-HSA- PTX-IR780. The results showed that the free drug generally exhibited higher toxicity than the nanosystems, both with and without PSN, probably due to the free drug internalization by cancer cells through passive diffusion. The enhancement of hyperthermia-induced “platelet bridge” drug accumulation was analyzed in vivo, with a great effect starting from heating at 45 °C (PTX release increases of 2.86×). In contrast with what was observed in vitro, tumor growth in vivo was reduced of about 90% compared to the control and of about 60% compared to the free drug. Furthermore, the diagnostic properties of PSN-HSA-PTX-IR780 were confirmed by fluorescence imaging in 4T1 mouse model. It is important to note that the developed system has shown its capabilities much better in vivo than in vitro, indicating how the behavior and performance of the nanoplatforms could drastically change in vivo, owing to the complexity of the physiological environment.

Virus-like particles (VLPs) are small non-infectious particles composed of viral capsid proteins without nucleic acid. These small particles show standard geometries and can be easily bio- and/or chemical-modified [70]. Moreover, VLPs can be loaded to deliver imaging agents and therapeutic molecules (e.g., chemotherapeutic drugs, small interfering RNA, aptamers, proteins and peptides) and can be modified with genetic engineering strategies to selectively target cells [76]. These particles have a wide-ranging application, including cancer therapies, immunotherapies, vaccines, cardiovascular therapies, gene therapies, imaging and theranostics [77].

Pitek et al. [78] exploited Tobacco Mosaic Virus (TMV) to realize serum albumin (SA)-coated NPs for theranostics. The authors used DOX as therapeutic drug and chelated gold (GdDOTA) as CA. SA-TMV NPs were tested in vitro in breast cancer cell lines (4T1 and MDA-MB-231), and in vivo in heterotopic model of breast cancer of human MDA-MB-231 cells in female NCR nu/nu mice, and in murine 4T1 cells in female Balb/c mice. The biodistribution in both mice models were assessed after 10 h p.i. with a high accumulation in liver and spleen (about 80% of the total fluorescence signal), due to these organs’ clearance functions. Tumor accumulation of bare TMV NPs was very low in both mouse models (about 1%) respect to SA-TMV NPs that showed an increased tumor accumulation (2% and 9% in the MDA-MB-231 and 4T1 models, respectively). This different accumulation could be related to the lower aggregation of SA-TMV compared to bare NPs owing to a blood circulation time 10 times higher, and to the interaction of albumin with gp60 and SPARC protein. An enhancement of therapeutic efficacy (tumor growth inhibition of about 50%) and an increase in survival rates in mice treated with SA-TMV NPs compared to free drug (80% and 25%, respectively) was observed. MR imaging technique was instead investigated in NCR nu/nu mice bearing PC3 xenograft by using GdDOTA properties as CA. The results showed a great spatial resolution and contrast for precise diagnosis in prostate tumors and a reduction of the signal after 24 h p.i., indicating efficacious biodegradation and clearance of the contrast agents from the body. This work is a good example of the very useful properties of albumin to cover NPs to increase their stability, biocompatibility, tumor accumulation and clearance.

Red blood cells membrane (RBC) can improve the pharmacokinetics, biodistribution and pharmacodynamics of therapeutics or diagnostics molecules [79] thanks to their high biomimetic properties, long half-life, and stability. Furthermore, they are easy to functionalize with specific ligands. Three types of carriers based on RBCs can be obtained: whole red blood cells loaded with drugs or NPs; NPs coated with RBC membrane; and nano-erythrosomes (NERs), which can be considered NPs derived from RBC [80].

Usually, whole RBCs, due to their micrometric size, are not able to reach solid tumors trough EPR effect or active targeting. To overcome this issue, Wang et al. [81] realized magnetic carriers RBCs-based for magnetic field (MF) enhanced drug delivery and imaging. RBCs were loaded with DOX and covered with oxide nanoparticles coated with chlorine e6 (Ce6). To enhance the stability, RBCs were further covered with PEG (DOX@RBC-IONP-Ce6-PEG). This system showed a long blood-circulation time (halftime of about 6 h), good DOX release profile (50% after 24 h and 80% after a week), and strong responses to an external MF. The therapeutic efficacy was tested in breast cancer cell line (4T1) by using RBC-IONP-Ce6-PEG or DOX@RBC- IONP-Ce6-PEG for 12 h of incubation with laser irradiation at 660 nm (5 mW cm^−2^ for 10 min). Low levels of toxicity were observed with RBC-IONP-Ce6-PEG or DOX@RBC- IONP-Ce6-PEG in dark conditions. On the other hand, when cells were irradiated an enhancement of toxicity was detected, owing to both enhanced DOX release and photodynamic properties. The magnetic targeting assessed in vitro revealed that, in presence of a magnetic field, RBCs-modified with IONPs increase their localization in the proximity of the magnet. The in vivo studies were performed by using Balb/c mice injected with 4T1 cells, and exploiting a magnet localized in tumor proximity for targeting. Mice were treated with both RBC-IONP-Ce6-PEG and IONP-Ce6-PEG and results showed both fluorescent and MR signal highly localized near to cancer tissues respect to healthy tissues. A reduction in off-target signal was detected when RBC-IONP-Ce6-PEG were used compared to IONP-Ce6-PEG, as consequence of highly blood circulation half-time of RBC-IONP-Ce6-PEG. The therapeutic efficacy in vivo was tested by using different RBC formulations with and without DOX, light irradiation, and external magnetic field. Of all the tested conditions, the use of DOX@RBC- IONP-Ce6-PEG with light irradiation and magnetic field showed the highest tumor growth reduction with no variation in mice weight. This is a good example of the exploitation of RBCs for theranostics that, despite their high size, can be effectively used both for imaging and therapy.

Another approach in the use of RBCs to obtain nanometric construct is that used by Burns et al. [82]. Optical nano constructs derived from erythrocytes doped with the NIR dye indocyanine green (ICG) (NETs) were investigated for their theranostic properties by exploiting ICG for NIR fluorescence imaging and for photodestruction both in vitro, in breast cancer cells (SKBR3), and in vivo in SKBR3 xenograft mice models. In vitro evaluation of cellular uptake was performed by incubating cells with free ICG (44 µM) vs NETs (ICG correspondent concentration of 36 µM) for 3 h. Cytofluorimetric analysis showed an ICG-signal 5-fold higher in NETs treated cells compared to free ICG treated cells. Furthermore, microscopic images revealed that NETs localized into lysosomes around the nucleus. In vitro, cytotoxic effect evaluation showed higher cytotoxicity in cells treated with NETs (70%) compared to free ICG (15%), after laser irradiation at 808 nm (0.68 W/cm^2^ for 15 min). Furthermore, the production of reactive oxygen species (ROS) and the apoptosis levels were assessed to investigate the cellular death mechanisms. The in vivo study by NIR fluorescence showed their high localization in tumor mass after 24 h post-injection. The relative tumor volume up to 16 days after NIR irradiation was measured, showing no tumor growth in mice treated with NETs, compared to control. This work shows the possibility to use RBCs membranes to design nanosystems with nanometric size without loss long half-life, and stability.

A summary of nanoparticles described in this review is reported in Table 1.

## 3. Conclusions

Nanotechnology applied to medicine (nanomedicine) led to the development of nanosystems that simultaneously exhibit diagnostic and therapeutic properties (theranostic nanoplatforms). These agents show several advantages, such as accumulation in tumor sites, versatility, and ease of functionalization, that can be exploited to realize “smart nanoplatforms” able to detect tumors, treat them, and monitor treatment response. Despite the advantages, the development, the design, and the fabrication still present many issues including biocompatibility, pharmacokinetics, in vivo targeting efficacy, and cost-effectiveness. Therefore, the most critical aspect in the future design of theranostic agents will be the optimization of some parameters such as size, shape, surface charge, composition, preparation protocols, decorating moieties, and drug loading and release rate. Currently, only few nanonoformulations are subject to clinical trials such as CriPec^®^ (Phase I clinical trial) and NBTXR3^®^ (Phase I/II clinical trials) while most of the advanced theranostic agents are confined to academic settings and present several clinical issues that must be resolved to consent the transition from the bench to bedside. The nanoparticles analyzed in this review represent some of the most innovative and recent systems for theranostic applications in cancer disease, collecting some of the most promising methods. Although they are not subjected to clinical trials, but only tested in cellular or mouse models, they could represent good candidates to be examined in clinical studies.

## Figures and Tables

**Table 1:** Resume of NPs for theranostic applications. not reported: n.r.

	Inorganic NPs				
	Size and Superficial Charge	Diagnosis	Therapy	Model	Ref.
Iron Oxide NPs	Size 45.7 nm ζ-potential n.r.	Magnetic Resonance Imaging	Magnetic resonance-guided focused ultrasound surgery	In vitro H460 cells In vivo H460 xenograft mice	[37]
Iron Oxide NPs	Size 50.8 nm ± 5.2 ζ-potential n.r.	Magnetic Resonance Imaging	Doxorubicin	In vitro 4T1 cells In vivo 4T1 xenograft mice	[38]
Iridium oxide NPs	Size 55.0 nm ζ-potential −0.40 mV	Fluorescence imaging	Doxorubicin Photothermal Therapy	In vitro HepG2 cells In vivo HepG2 xenograft mice	[40]
MOF Fe_3_O_4_@UiO-66	Size 241.5 nm ± 28.5 ζ-potential −25.7 mV ± 5.2	Magnetic Resonance Imaging	Doxorubicin	In vitro HeLa cells In vivo HeLa-tumor bearing mice	[44]
MOF NaGdF_4_:Yb/Er@MIL-53(Fe)	Size 245 nm ± 5.0 ζ-potential n.r.	Magnetic Resonance Imaging	Doxorubicin	In vitro B16−F10 and HEK293 cells	[45]
Gold NPs	Size 26.5 nm ± 1.1 ζ-potential n.r.	Fluorescence imaging	Photodynamic Therapy	In vitro PC-3 cells In vivo PC-3 xenograft mice	[49]
Gold NPs	Size 390.0 nm ζ-potential n.r.	Photoacoustic imaging	Photothermal Therapy	In vitro U-87MG cells In vivo U-87MG xenograft mice	[50]
Lanthanide-doped NPs NaYF4:Yb, Tm@NaYF4:Eu	Size 141.9 nm ζ-potential −20.2 mV	Upconversion luminescence imaging	Photodynamic Therapy	In vitro AGS cells In vivo AGS xenograft mice	[56]
Lanthanide-doped NPs NaLuF4	Size 20 × 130 nm ζ-potential n.r.	NIR-II imaging	Photothermal therapy	In vitro HeLa cells In vivo HCT 116 xenograft mice and LLC	[55]
Silicon-based	Size 407.0 nm ± 29.0 ζ-potential −17.0 mV ± 2.16	Photoacoustic Imaging	Photothermal therapy Doxorubicin	In vitro MCF10a and HS578T cells	[20,57,58]
Silicon-based	Size 13.5 nm ζ-potential n.r.	PET imaging	Radiotherapy	In vivo 4T1 tumor-bearing mice	[59]
	**Organic NPs**				
PLGA-based NPs	Size 282.1 nm ± 6.2 ζ-potential −9.7 mV ± 1.4	Magnetic Resonance Imaging	Radio frequency hyperthermia Docetaxel	In vitro MCF7 cells In vivo S180 xenograft mice	[65]
PLGA-based NPs	Size 185.1 nm ± 3.3 ζ-potential −1.2 mV ± 0.7	Photoacoustic imaging	Photothermal therapy	In vitro MDA-MB-231 cells In vivo MDA-MB-231 xenograft mice	[26]
PLGA-based NPs	Size 248.3 nm ζ-potential −14.7 mV	Magnetic Resonance Imaging Dual-modal ultrasound	Photothermal therapy	In vitro SKBR3 and MDA-MB-231 cells	[67]
Chitosan-based NPs	Size 184.3 nm ± 4.4 ζ-potential + 17.33 mV ± 1.5	Magnetic Resonance Imaging	Doxorubicin	In vitro C6 cells	[68]
Chitosan-based NPs	Size 92.2 nm ζ-potential + 24.0 mV	Fluorescence imaging	Nucleic acid	In vitro HeLa cells	[69]
Liposomes-based NPs	Size 95.0 nm ζ-potential n.r.	Positron Emission Tomography Fluorescence Photoacoustic imaging	Photodynamic therapy AQ4N	In vivo 4T1 Balb/c mice	[72]
Liposomes-based NPs	Size 150–300 nm ζ-potential + 13.2 mV	Fluorescence imaging	Photothermal therapy Doxorubicin	In vitro MDA-MB-231 and 4T1 cells In vivo 4T1 Balb/c mice	[73]
Albumin NPs	Size 142.2 nm ± 4.86 ζ-potential −30 mV	Fluorescence imaging	Photodynamic therapy Photothermal therapy Paclitaxel	In vitro 4T1 cells In vivo 4T1 Balb/c mice	[75]
Virus like-NPs	Size 212.0 nm ± 3.40 ζ-potential n.r.	Fluorescence imaging	Doxorubicin	In vitro 4T1and MDA-MB-231 cells In vivo 4T1 Balb/c mice MDA-MB-231 and PC-3 xenograft mice	[78]
Red Blood cells-based NPs	Size about 7 µm ζ-potential n.r.	Magnetic Resonance Imaging Fluorescence imaging	Photodynamic therapy Doxorubicin	In vitro 4T1 cells In vivo 4T1 Balb/c mice	[81]
Red Blood cells-based NPs	Size 79.0 nm ζ-potential n.r.	Fluorescence imaging	Photodestruction	In vitro SKBR3 cells In vivo SKBR3 xenograft mice	[82]
